# Tracking of apolipoprotein B levels measured in childhood and adolescence: systematic review and meta-analysis

**DOI:** 10.1007/s00431-023-05350-0

**Published:** 2023-12-05

**Authors:** Oliver Stanesby, Zhen Zhou, Ricardo Fonseca, Tetsuhiro Kidokoro, Petr Otahal, Brooklyn J. Fraser, Feitong Wu, Markus Juonala, Jorma S. A. Viikari, Olli T. Raitakari, Grant R. Tomkinson, Costan G. Magnussen

**Affiliations:** 1 Menzies Institute for Medical Research, University of Tasmania, Hobart, Australia; 2 Baker Heart and Diabetes Institute, Melbourne, Australia; 3 School of Public Health and Preventive Medicine, Monash University, Melbourne, Australia; 4 Primary Health Tasmania, Hobart, Australia; 5 Research Institute for Health and Sport Science, Nippon Sport Science University, Tokyo, Japan; 6 Alliance for Research in Exercise, Nutrition and Activity (ARENA), Allied Health and Human Performance, University of South Australia, Adelaide, Australia; 7 Baker Department of Cardiometabolic Health, Faculty of Medicine, Dentistry and Health Sciences, University of Melbourne, Melbourne, Australia; 8 Department of Medicine, University of Turku, Turku, Finland; 9 Division of Medicine, Turku University Hospital, Turku, Finland; 10 Research Centre of Applied and Preventive Cardiovascular Medicine, University of Turku, Turku, Finland; 11 Centre for Population Health Research, University of Turkuand, Turku University Hospital, Turku, Finland; 12 Department of Clinical Physiology and Nuclear Medicine, Turku University Hospital, Turku, Finland

**Keywords:** Adolescent, Apolipoproteins, Child, Meta-analysis, Systematic review

## Abstract

To quantify the tracking of apolipoprotein B (apoB) levels from childhood and adolescence and compare the tracking of apoB with low-density lipoprotein (LDL) cholesterol, a systematic search of MEDLINE, Embase, Web of Science, and Google Scholar was performed in October 2023 (PROSPERO protocol: CRD42022298663). Cohort studies that measured tracking of apoB from childhood/adolescence (< 19 years) with a minimum follow-up of 1 year, using tracking estimates such as correlation coefficients or tracking coefficients, were eligible. Pooled correlations were estimated using random-effects meta-analysis. Risk of bias was assessed with a review-specific tool. Ten studies of eight unique cohorts involving 4677 participants met the inclusion criteria. Tracking of apoB was observed (pooled *r* = 0.63; 95% confidence interval [CI] = 0.53–0.71; *I*^2^ = 96%) with no significant sources of heterogeneity identified. Data from five cohorts with tracking data for both lipids showed the degree of tracking was similar for apoB (pooled *r* = 0.59; 95% CI = 0.55–0.63) and LDL cholesterol (pooled *r* = 0.58; 95% CI = 0.47–0.68). Study risk of bias was moderate, mostly due to attrition and insufficient reporting.

*Conclusion*: ApoB levels track strongly from childhood, but do not surpass LDL cholesterol in this regard. While there is strong evidence that apoB is more effective at predicting ASCVD risk than LDL cholesterol in adults, there is currently insufficient evidence to support its increased utility in pediatric settings. This also applies to tracking data, where more comprehensive data are required.

**Table:** 

**What is known:** *• Apolipoprotein B is a known cause of atherosclerotic cardiovascular disease.* *• Apolipoprotein B levels are not typically measured in pediatric settings, where low-density lipoprotein cholesterol remains the primary lipid screening measure.*	
**What is new:** *• This meta-analysis of 10 studies showed apolipoprotein B levels tracked strongly from childhood but did not exceed low-density lipoprotein cholesterol in this regard.* *• More comprehensive tracking data are needed to provide sufficient evidence for increased utility of apolipoprotein B in pediatric settings.*

**Supplementary Information:**

The online version contains supplementary material available at 10.1007/s00431-023-05350-0.

## Introduction

The concentration of cholesterol contained in lipids such as low-density lipoprotein (LDL) and non-high-density lipoprotein (non-HDL) cholesterols, in addition to the concentration of triglycerides, has a long history of association with atherosclerotic cardiovascular disease (ASCVD). As such, these lipids tend to form a cornerstone of screening and treatment for the reduction of ASCVD in adult settings [1–3]. Although the concentration of cholesterol and triglycerides in each of these conventional lipids and their subtypes can differ substantially, each of these circulating atherogenic lipoproteins contains a molecule of apolipoprotein B-100 (apoB) [4]. This hepatically produced protein is a key component of very-low-density lipoprotein, LDL, intermediate-density lipoprotein particles and is present in lipoprotein(a) [5]. Most evidence from large and rigorously performed studies shows that the total number of apoB-containing lipoprotein particles, not cholesterol or triglyceride concentration, is the key lipid determinant of ASCVD [6–9], shorter lifespan, and type 2 diabetes [10]. Hence, it is proposed that apoB combines information from conventional lipids into a singular index quantifying ASCVD risk [4, 11–13].

In the USA, population-wide screening for conventional lipid levels targeting pediatric ASCVD risk reduction is recommended at ages 9–11 years and again at 17–21 years [14]. While pediatric screening for lipid levels is controversial [15, 16], the persistence of these lipid levels (tracking) into later life partly informs the case for screening recommendations [14, 17–19]. Given the emerging role of apoB as the key lipid determinant in ASCVD, we performed a systematic review and meta-analysis to examine the degree to which apoB levels track from childhood and adolescence into later life and how this compares to LDL cholesterol.

## Methods

The review protocol was registered with the International Prospective Register of Systematic Reviews (PROSPERO ID: CRD42022298663). It was designed, conducted, and reported in alignment with systematic review best practices, including the Preferred Reporting Items for Systematic Reviews and Meta-analyses (PRISMA) Statement 2020 (Online Resource Table 1), Cochrane Handbook, Embase Guide, and university library guides [20–24].

**Table 1:** Eligibility criteria

*Inclusion criteria*	
• Studies in humans. • Cohort study (follow-up, longitudinal, prospective, or retrospective). • Sample/subsample aged < 19 y at baseline (determined by sample/subsample mean/median age or midpoint of age range if mean/median unavailable) from the general population. • ApoB levels measured at baseline in childhood or adolescence (via laboratory test/assay of serum/plasma/blood, or calculation from other lipid and lipoprotein levels, or extraction from medical record) and again ≥ 1 y later. • Quantitative analysis of the degree of tracking of apoB levels from childhood and adolescence (baseline) into later life (follow-up), determined as a correlation coefficient (e.g., Pearson, Spearman, Kendall, including partial) or tracking coefficient (via generalized estimating equations or multivariate analysis of a banded model). • Full-text original empirical research journal articles that are published or in press. • No date restrictions will be imposed.	

*Exclusion criteria*	
• Studies specifically focusing on targeted populations (e.g., familial hypercholesterolemia, positive family history of premature coronary heart disease, those with clinical obesity). • All study types other than cohort (e.g., randomized controlled trial, experimental, case–control, cross-sectional, review, qualitative, non-empirical).	

Eligible publications must bear all inclusion criteria and no exclusion criteria

*apoB* apolipoprotein B

### Eligibility criteria

Eligible studies were cohort studies that measured the degree of tracking of apoB levels from childhood or adolescence (age < 19 years) into later life (follow-up period ≥ 1 year) with a correlation coefficient or a tracking coefficient (which is comparable but not equivalent to a correlation [25]). A minimum follow-up period of 1 year was chosen because it typically coincides with recommendations for routine (yearly) clinical assessment of ASCVD risk factors such as body mass index and blood pressure in the pediatric setting [26, 27]. The full eligibility criteria are described in Table 1.

### Information sources

We identified studies by searching electronic databases, reference lists, topical reviews, and authors’ personal databases. MEDLINE (via Ovid, 1946 to 13 October 2023) Embase (via Ovid, 1974 to 13 October 2023), Web of Science (Core Collection, 1945 to 16 October 2023), and Google Scholar (first 200 results on 14 January 2022 and 16 October 2023) were searched by one researcher (OS). We applied Bramer et al.’s recommended optimal combination of electronic databases [28] and designed the search strategy in consultation with University of Tasmania library staff experienced in electronic literature searching.

### Search strategy

The search strategy aimed to capture cohort studies that mentioned apolipoproteins, childhood or adolescence, and tracking. Subject headings, keywords, titles, and abstracts (full text for Google Scholar) were searched. The full search strategy (terms and parameters) for each database is described in Online Resource Table 2.

### Study selection

Studies from our search were imported to Covidence online software (Veritas Health Innovation, Melbourne, VIC, Australia) and de-duplicated. Two researchers (OS, ZZ) independently screened the titles and abstracts against the eligibility criteria and then the full texts for those that passed title/abstract screening, with conflicts resolved via an adjudicating vote from a third researcher (CGM). Two researchers (RF, TK) assisted with screening full texts in languages other than English (four Spanish, one Japanese).

### Data extraction

Relevant data were extracted (including supplemental material if necessary) by one researcher (OS or RF). Information that was not reported (missing data) was sought in other published material about the studies. A second researcher (ZZ or OS) verified the data extracted from each study. Discrepancies were reviewed by three researchers (OS, ZZ, CGM) and corrections were made if necessary. Extracted data were recorded and stored in Covidence.

### Data items and effect measures

The following information was extracted about each study: study name, study design, and country where data were collected. The following information was extracted about each eligible tracking estimate: type of apolipoprotein/lipid, effect estimate, tracking effect measure, variables used for adjustment, sample size, sex, ethnicity, age at baseline, length of follow-up, fasting status at baseline and follow-up as defined by the authors, number of serum measurements used to derive levels at baseline and follow-up, and measurement methods.

### Risk of bias assessment

A modified version of the Newcastle–Ottawa Scale for assessing the quality of cohort studies was used to assess the risk of bias of the studies (Online Resource Methods 1) [29]. Two researchers (OS, ZZ) independently assessed the risk of bias of the studies, with another researcher (RF) assisting with the assessments of materials in languages other than English. Discrepancies were reviewed and resolved by three researchers (OS, ZZ, CGM).

### Synthesis methods

Descriptive statistics were used to summarize and compare the characteristics of the included studies and cohorts. Discrete variables were summarized as counts (percentages) and continuous variables as mean (standard deviation [SD], range). We used the DerSimonian-Laird random-effects meta-analysis to estimate the tracking of apoB from childhood and adolescence, quantified as pooled correlation or tracking coefficients, weighted by the standard error of the effect estimate. Stratified random-effects meta-analysis compared the tracking of apoB and LDL cholesterol levels among studies with data available on both. Estimates were Z-transformed for meta-analysis and back-transformed for interpretation. Each cohort contributed up to one observation per meta-analysis; if an overall effect estimate for the cohort was not reported, the weighted mean among the applicable subgroup estimates was used. Forest plots visualized cohort-level and pooled effect estimates and their 95% confidence intervals (CIs). Statistical (between-cohort) heterogeneity was quantified by the *I*^2^ statistic and interpreted according to the Cochrane Handbook [30]. Potential sources of heterogeneity (sex, length of follow-up, age at baseline, sex, and age at baseline strata) were examined by exploratory stratified random-effects meta-analysis. Analyses were performed with Stata version 17 (StataCorp, College Station, TX, USA). Statistical significance level was set at *p* < 0.05.

### Reporting bias assessment

Publication bias was visually examined by funnel plots, and the pooled coefficient including potentially missing studies was estimated with nonparametric restricted maximum likelihood trim-and-fill analysis.

### Certainty of evidence assessment

We used the Grading of Recommendations Assessment, Development, and Evaluation (GRADE) approach, a widely endorsed method for grading the quality of evidence and the strength of recommendations, to assess the certainty of the evidence [31, 32].

## Results

### Study selection

**Fig. 1:**
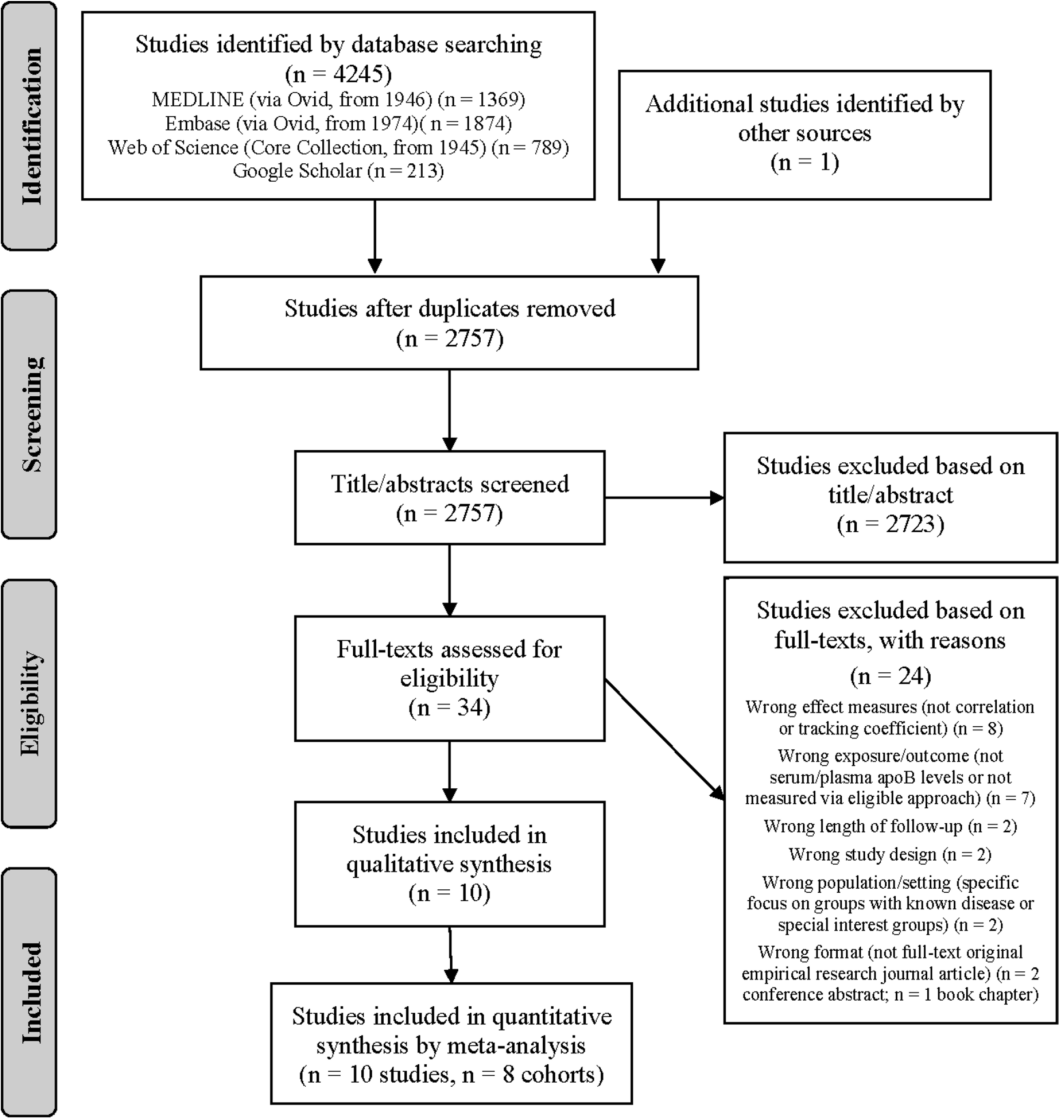
Phases of study selection. Databases searched: 16 October 2023; figure adapted from the PRISMA statement [20]; apoB: apolipoprotein B

The flow of studies through each phase of selection and screening is shown in Fig. 1. After removal of 1489 duplicates, 2757 unique studies were screened for eligibility, 2723 of these were excluded during title and abstract screening, and the full texts of the remaining 34 studies were assessed. Another 24 studies were ineligible during full-text screening; the most frequent reasons for exclusion were did not use required effect measures (*n* = 8) and did not measure serum/plasma apoB levels (*n* = 7). Ten studies were included [33–42].

### Study characteristics

This review extracted apoB and LDL cholesterol tracking estimates from 10 studies of eight unique cohorts of 40 to 1758 participants (totaling 4677 unique participants). The studies and cohorts are summarized in Table 2 and extracted estimates are described in Online Resource Table 3. Data was collected in Europe and the USA between 1974 and 2020. Most studies reported multiple eligible tracking estimates because analyses were often stratified by subgroups (e.g., sex, age at baseline, length of follow-up, timepoint pairs). The results of six cohorts were stratified by sex [33, 34, 36, 38–40]. Age at baseline ranged from 0.0 to 16.0 years and length of follow-up ranged from 1.0 to 7.4 years, and tracking to adulthood (age ≥ 19 years at follow-up) was assessed in two cohorts [33, 39]. One study reported estimates adjusted for age, height, and ponderal index [33]. The adjusted estimates were pooled with unadjusted estimates because the authors reported they were essentially the same as the unreported unadjusted estimates and excluding the adjusted estimates did not change the meta-analysis results. The number of measurements per person varied across the included studies, but no studies derived baseline levels or follow-up levels from multiple blood draws that were averaged.

### Risk of bias

Risk of bias scores are summarized in Table 2. On average, the studies were assessed as having a moderate risk of bias, with a high risk of bias due to attrition, measurement, and potential conflicts of interest, and a moderate risk of bias due to sample selection and confounding. Insufficient reporting contributed to the risk of bias, with studies providing insufficient detail for 39% of the risk of bias items (60% for representativeness of sample, 55% for adequacy of follow-up, 55% for lipid measurement protocols, 30% for number of measurements at timepoints, 10% for fasting status, 18% for confounding, 70% for conflicts of interest).

**Table 2:** Characteristics of the eight cohorts (from 10 studies)

	**Bogalusa Heart Study**	**Cardiovascular Risk in Young Finns Study**	**Four Provinces Study**	**Life Child Study**	**Madrid Study (no official name)**	**Rivas-Vaciamadrid Study**	**Tallinn Young Family Study**	**Umeå Study (no official name)**	**Total**
Publications included, *n*	2	1	1	1	1	2	1	1	10
									**Mean (SD)**
Sample size at follow-up, *n*	1758	333	385	1313	237	493	40	118	585 (616)
Risk of bias (higher score = higher risk of bias)									
Selection bias, 0–1 score	0	0	1	1	0	1	1	1	0.6 (0.5)
Attrition bias, 0–1 score	1	1	1	1	0	1	1	1	0.9 (0.4)
Measurement bias, 0–3 score	1	2	2	1	2	2.6^a^	1	3	1.8 (0.8)
Confounding bias, 0–2 score	1	1	2	1	1	1.3^a^	1	0	1.0 (0.5)
Conflicts of interest bias, 0–1 score	1	1	0	0	1	1	1	0	0.6 (0.5)
Total risk of bias, 0–1 score	4	5	6	4	4	6.9^a^	5	5	5.0 (1.0)
									***n* (%)**
Lipids measured									
apoB	✓	✓	✓	✓	✓	✓	✓	✓	8/8 (100%)
LDL cholesterol	✓	×	✓	× ^b^	✓	✓	×	✓	5/8 (63%)
Tracking measure									
Spearman correlation coefficient	×	✓	×	×	×	×	×	×	1/8 (13%)
Partial Spearman correlation coefficient	✓	×	×	×	×	×	×	×	1/8 (13%)
Pearson correlation coefficient	×	×	×	✓	×	✓	✓	✓	4/8 (50%)
Partial Pearson correlation coefficient	×	×	×	×	×	×	×	×	0/8 (0%)
Unclear type of correlation coefficient	✓	×	✓	×	✓	×	×	×	3/8 (38%)
Sex									
Males and females combined	✓	×	×	✓	✓	✓	✓	×	5/8 (63%)
Males	✓	✓	✓	×	✓	✓	×	✓	6/8 (75%)
Females	✓	✓	✓	×	✓	✓	×	✓	6/8 (75%)
Baseline age^c^									
< 5 y	✓	×	×	✓	×	×	✓	✓	4/8 (50%)
5–9 y	✓	×	✓	✓	✓	✓	×	×	5/8 (63%)
10–14 y	✓	×	×	✓	✓	×	×	×	3/8 (38%)
15–18 y	✓	✓	×	✓	×	×	×	×	3/8 (38%)
Length of follow-up^c^									
< 5 y	✓	✓	×	✓	×	✓	×	✓	5/8 (63%)
5–9 y	×	×	✓	×	✓	✓	✓	×	4/8 (50%)
≥ 10 y	×	×	×	×	×	×	×	×	0/8 (0%)
Follow-up in adulthood (mean age ≥ 19 y)	✓	×	×	×	✓	×	×	×	2/8 (25%)
Fasted at baseline serum measurement	✓	✓	✓	✓	✓	✓	×	×	6/8 (75%)
Unfasted at baseline serum measurement	×	×	×	✓	×	×	✓	✓	3/8 (38%)
Fasted at follow-up serum measurement	✓	✓	✓	✓	✓	✓	✓	✓	8/8 (100%)
Unfasted at follow-up serum measurement	×	×	×	✓	×	×	×	✓	2/8 (25%)

Sum of rows may exceed *n* = 8 or 100% because cohorts with stratified analyses may apply to multiple categories

*apoB* apolipoprotein B, *LDL* low-density lipoprotein, *IQR* interquartile range, *SD* standard deviation

^a^Cohort average across publications, weighted by sample size

^b^Measured LDL-C but samples not equivalent to apoB

^c^Mean/median or midpoint of range if mean/median not reported

### Results of syntheses

**Fig. 2:**
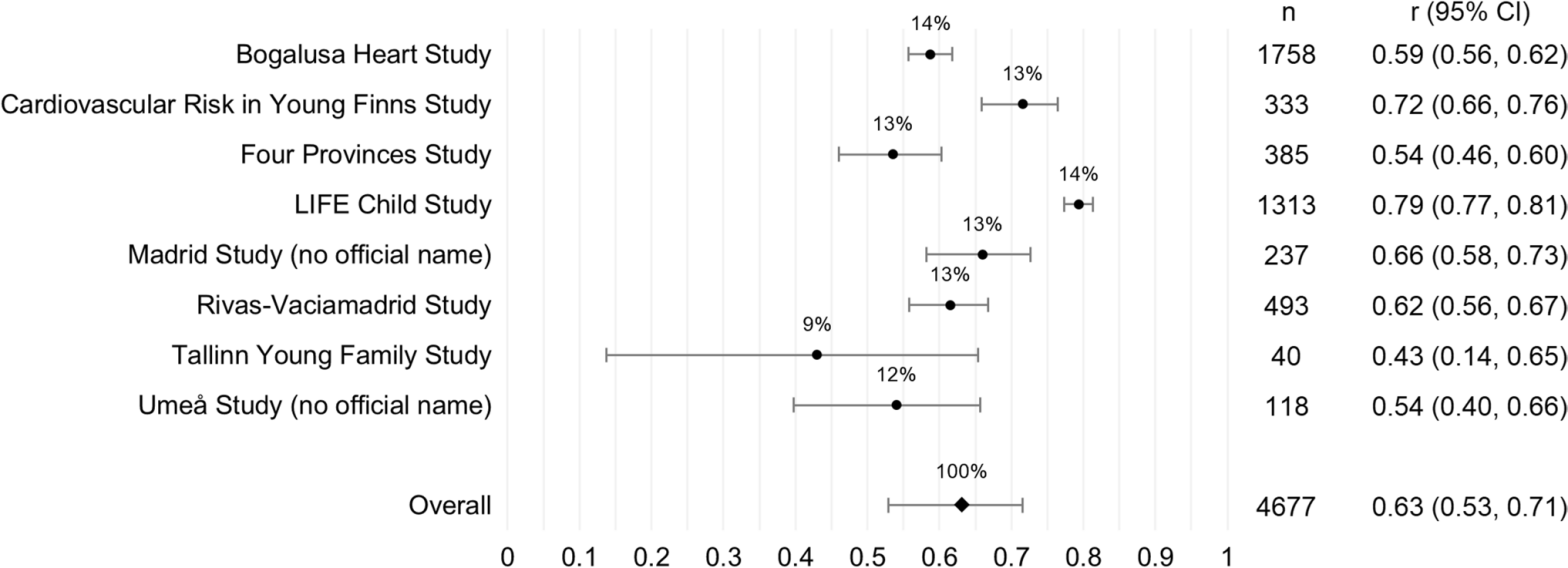
Random-effects meta-analysis of tracking of apoB from childhood/adolescence to at least 1 year later in life. Effect estimates (*r*) pooled across cohorts using DerSimonian-Laird random-effects model; *I*^2^ = 96% (95% CI = 62%, 99%); weight (%) indicated above effect estimates; 95% confidence interval (CI) represented by capped lines; apoB: apolipoprotein B

Collectively, there was a positive correlation for tracking of apoB levels (*r* = 0.63, 95% CI = 0.53, 0.71; Fig. 2). An estimated 40% (*r*^2^) of variation in later-life apoB levels was explained by variation in childhood/adolescence apoB levels. There was considerable heterogeneity between cohorts (Fig. 2). In exploratory subgroup analyses (not tabulated), neither sex, length of follow-up, age at baseline, nor sex and age at baseline stratification (pre-pubertal males, pre-pubertal females, peri-pubertal males, peri-pubertal females) explained the observed heterogeneity.

Meta-analysis of tracking of apoB to adulthood was not possible due to an insufficient number of cohorts (*n* = 2). Correlation coefficients ranged from 0.20 to 0.69 (cohort mean *r* = 0.45 and 0.69 for the cohorts) for tracking from late adolescence (14–18 years) to early adulthood (19–22 years) [33, 39].

**Fig. 3:**
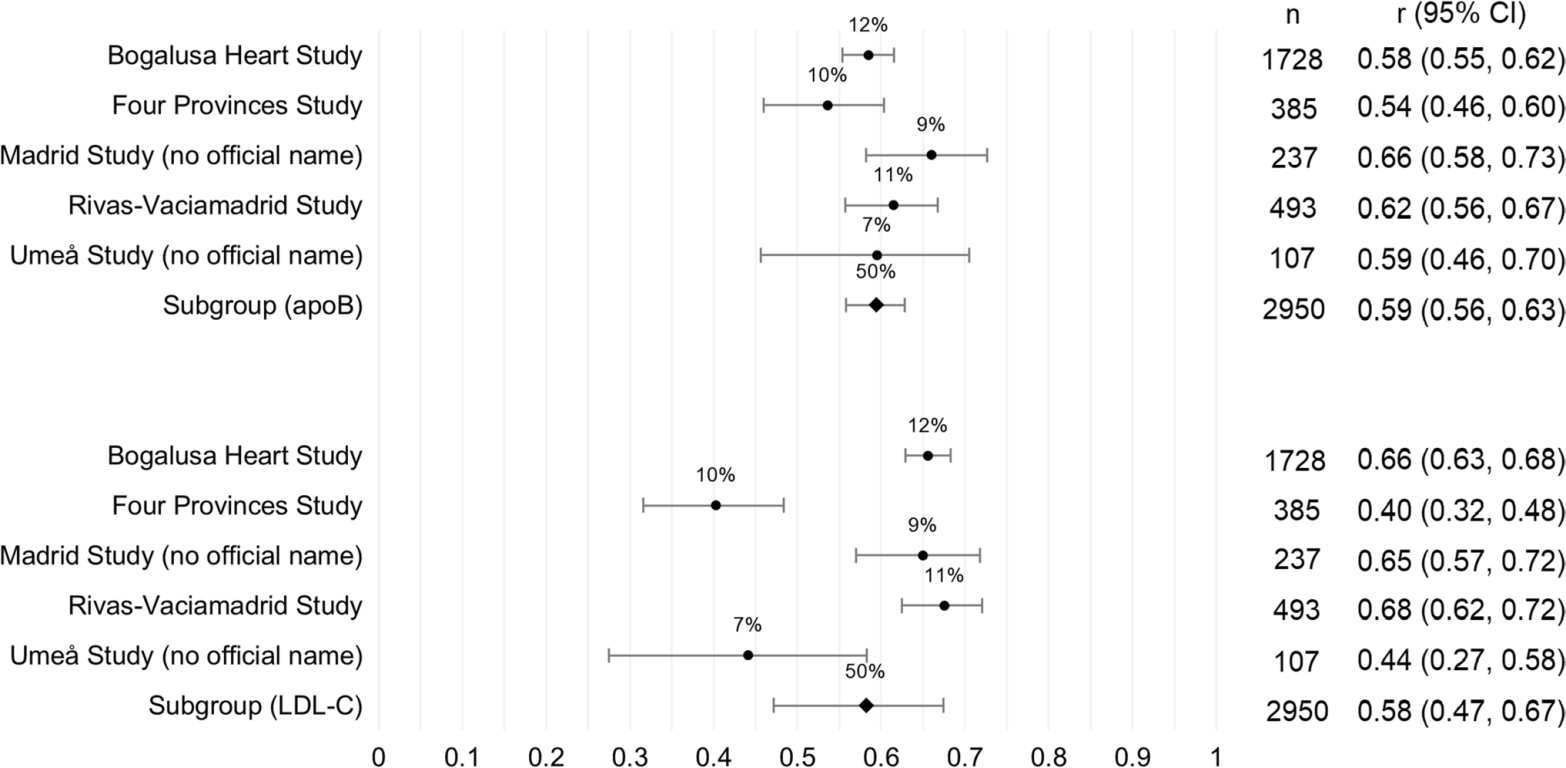
Stratified random-effects meta-analysis of tracking of apoB and LDL cholesterol from childhood/adolescence to at least 1 year later in life. Effect estimates (*r*) pooled across cohorts using DerSimonian-Laird random-effects model; apoB subgroup *I*^2^ = 38%; LDL cholesterol subgroup *I*^2^ = 92%; weight (%) indicated above effect estimate; 95% confidence interval (CI) represented by capped lines; apoB: apolipoprotein B; LDL: low-density lipoprotein

No difference in the degree of tracking of apoB and LDL cholesterol was found across the five cohorts that reported the tracking of both lipids (Fig. 3).

### Reporting biases

**Fig. 4:**
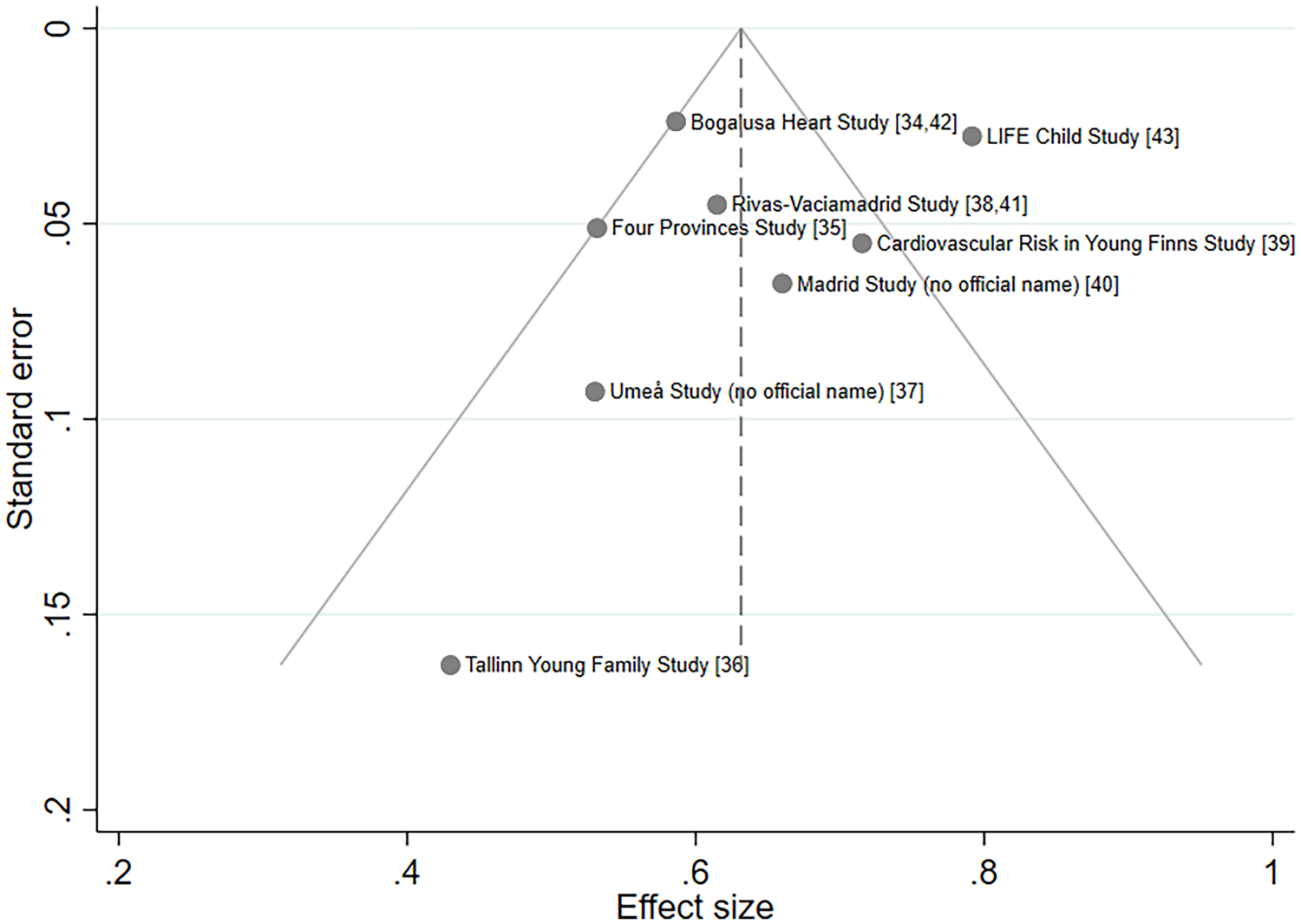
Funnel plot of the effect estimate (*r*) and standard error (*se*_*r*_) of tracking of apoB from childhood/adolescence to at least 1 year later in life. The circles represent the cohort-specific coefficients, the vertical line represents the pooled coefficient, and the dashed triangular region represents the boundary within which 95% of studies are expected to fall in the absence of both biases and heterogeneity; apoB: apolipoprotein B

Despite insufficient cohorts (*n* < 10) to statistically quantify the presence of publication bias in the reporting of apoB tracking, there was some visual evidence of asymmetry in the funnel plot, with a deficit of small studies reporting a large effect size (Fig. 4). Imputation of two potentially missing studies increased the estimated degree of apoB tracking by 5% (pooled *r* = 0.66; 95% CI = 0.59, 0.72).

## Discussion

This meta-analysis found a single measure of apoB from childhood or adolescence tracks strongly into later life. A subanalysis showed apoB and LDL cholesterol tracked similarly. Applying the GRADE approach to the primary research question [31, 32], we are moderately confident in the effect estimates. The true tracking of apoB is likely to be close to the estimate of the effect, but there is a possibility that it is substantially different due to risk of bias, heterogeneity, inconsistent use of cut-offs for risk classification, and absence of data on tracking to adulthood.

The included studies had moderate risk of bias, with insufficient detail on lipid measurement protocols, sample selection, and conflicts of interest, but no visual evidence of publication biases was detected. The main meta-analysis showed considerable variation between studies which was not explained by differences in the length of follow-up or the sex or age of the samples at baseline. Attrition was a common source of risk of bias, with little consideration of potential differential loss to follow-up or use of statistical approaches to account for it.

This review considered tracking of continuous lipid levels via correlation, describing how relative rankings based on a lipid level at one timepoint can predict those at a later timepoint [38, 43, 44]. It did not cover the tracking of categorical risk factor levels which examines the degree to which risk *classification* remains stable over time—information that is much more clinically useful [45]. The focus on tracking of continuous levels was necessary to allow meta-analysis because cut-offs used to denote high risk wary widely among studies (studies either defined high risk arbitrarily as the upper quartile or quintile of the sample distribution or used different pre-specified thresholds [33–35, 37–40]) which might reflect a lack of consensus cut-offs for apoB, particularly prior to the 2011 guidelines [14]. Another gap in the literature is the absence of data on the tracking of apoB from childhood to middle and late adulthood, when clinical ASCVD typically presents [46, 47]. Also, while screening guidelines recommend calculating levels from two lipid profile measurements to limit measurement error due to within-person variability [17, 48–50], no studies adhered to this practice. Thus, the true degree of tracking may be higher in practice where current guidelines recommend multiple measurements than estimated in this meta-analysis. There is also limited tracking data that considers sex and age, or reliable assessment of sexual maturation status, at baseline, which is needed to consider any potential influence of changes in lipid levels that occur with puberty on tracking [51, 52].

The heterogeneity analyses, risk of bias assessment, and absence of data for risk classification stability and tracking to adulthood suggest more data are needed to fully assess the case for screening based on tracking. The availability of standardized apoB methodology [53], valid estimation of apoB into adulthood from standard lipid measurements (total cholesterol, HDL cholesterol, triglycerides) [54], standardized pediatric cut-offs to denote high risk [14, 17–19], and the likely reduction in risk of bias that could be attained by simple reporting of key details and consideration of differential loss to follow-up mean that the *more data are needed* should be readily obtainable from pre-existing cohort studies.

The screening, treatment, and clinical management of pediatric lipid levels to reduce ASCVD risk have predominantly revolved around LDL cholesterol, estimated from standard lipid panel assays of total cholesterol, HDL cholesterol, and triglycerides [14, 16, 55, 56]. The extensive use of LDL cholesterol as the primary outcome in clinical trials, combined with its long-standing familiarity among physicians, grants it significant inertia in clinical practice. For any emerging marker, whether it be apoB or non-HDL cholesterol, to gain widespread clinical acceptance, it would likely need to outperform LDL cholesterol in terms of clinical benefits or have considerable practical advantages. A growing body of evidence, primarily stemming from adult populations, suggests that apoB and non-HDL cholesterol may offer superior risk prediction capabilities compared to or alongside other lipids and lipoproteins and can be measured without the need to fast [6–9, 55, 57–62]. While childhood data regarding apoB’s and non-HDL cholesterol’s utility remains limited, preliminary findings hint at their potential to bolster current and future ASCVD risk prediction [63–68], especially among those with obesity-associated dyslipidemia characterized by hypertriglyceridemia and low HDL cholesterol [69]. However, the adoption of apoB faces challenges. Notably, the adherence of pediatricians to existing guidelines in the USA where universal lipid screening in children is endorsed is disappointingly low [70, 71]. Introducing a new or additional measure with yet-to-be-defined reference values and concerns over added costs and standardization—though these have largely been addressed [72, 73]—might exacerbate the reluctance and difficulties in implementation among physicians [71, 73, 74]. These concerns may be less pertinent for non-HDL cholesterol, which can be accurately calculated from a standard lipid panel and is increasingly favored for ASCVD health and risk reduction [6–9, 14, 75, 76]. Decisive factors in the uptake of any lipid measurement(s) in pediatric care will hinge on its predictive utility and the subsequent physician education around it [71]. Although current evidence is preliminary, direct evidence with clinical outcomes might soon emerge from the International Childhood Cardiovascular Cohort (i3C) Consortium [77].

### Strengths and limitations

This systematic review adheres to best practices and was developed in consultation with leading experts [20–23]. It presents the most comprehensive synthesis and only meta-analysis of apoB tracking data to date. However, cohorts contributing data to this review were from high-income countries of Estonia, Finland, Sweden, Spain, and the USA and therefore may not be generalizable to low- or middle-income countries or regions outside of Europe and North America [78].

## Conclusions

ApoB levels exhibit strong tracking from childhood, though they do not surpass LDL cholesterol in this regard. Although there is a strong evidence base in adults for apoB’s enhanced ASCVD risk prediction capabilities independent of, or in addition to, LDL cholesterol, evidence for increased utility of apoB in pediatric settings is currently too few. This also applies to tracking data, where more comprehensive data are needed.

### Supplementary Information

Below is the link to the electronic supplementary material.

Supplementary file1 (DOCX 63 KB)

## Data Availability

The datasets generated and analyzed during the current study are available in the University of Tasmania Research Data Portal and Research Data Australia repository (Stanesby O, Magnussen C (2023) Data from: Dataset - Tracking of apolipoprotein B levels measured in childhood and adolescence: systematic review and meta-analysis. 10.25959/vrta-vt46).

## Declarations

### Disclaimer

The contents of the published material are solely the responsibility of the individual authors and do not reflect the views of the NHMRC.
