# Correction to: Tracking of apolipoprotein B levels measured in childhood and adolescence: systematic review and meta-analysis

**DOI:** 10.1007/s00431-024-05445-2

**Published:** 2024-01-31

**Authors:** Oliver Stanesby, Zhen Zhou, Ricardo Fonseca, Tetsuhiro Kidokoro, Petr Otahal, Brooklyn J. Fraser, Feitong Wu, Markus Juonala, Jorma S. A. Viikari, Olli T. Raitakari, Grant R. Tomkinson, Costan G. Magnussen

**Affiliations:** 1 Menzies Institute for Medical Research, University of Tasmania, Hobart, Australia; 2 Baker Heart and Diabetes Institute, Melbourne, Australia; 3 School of Public Health and Preventive Medicine, Monash University, Melbourne, Australia; 4 Primary Health Tasmania, Hobart, Australia; 5 Research Institute for Health and Sport Science, Nippon Sport Science University, Tokyo, Japan; 6 Alliance for Research in Exercise, Nutrition and Activity (ARENA), Allied Health and Human Performance, University of South Australia, Adelaide, Australia; 7 Baker Department of Cardiometabolic Health, Faculty of Medicine, Dentistry and Health Sciences, University of Melbourne, Melbourne, Australia; 8 Department of Medicine, University of Turku, Turku, Finland; 9 Division of Medicine, Turku University Hospital, Turku, Finland; 10 Research Centre of Applied and Preventive Cardiovascular Medicine, University of Turku, Turku, Finland; 11 Centre for Population Health Research, University of Turkuand, Turku University Hospital, Turku, Finland; 12 Department of Clinical Physiology and Nuclear Medicine, Turku University Hospital, Turku, Finland

**Correction to: European Journal of Pediatrics** 10.1007/s00431-023-05350-0

The Data statement was missing the reference link and should have read "The datasets generated and analyzed during the current study are available in the University of Tasmania Research Data Portal and Research Data Australia repository (Stanesby, O, Magnussen, C (2023) Data from: Dataset - Tracking of apolipoprotein B levels measured in childhood and adolescence: systematic review and meta-analysis. 10.25959/vrta-vt46).”

The original article has been corrected.

