# Supplementary material for: Tracking of apolipoprotein B levels measured in childhood and adolescence: systematic review and meta-analysis
Source: Eur J Pediatr. 2023 Dec 5;183(2):569–80. doi: 10.1007/s00431-023-05350-0 (PMC10912277; doi:10.1007/s00431-023-05350-0)
Supplement: Supplementary file 1 — Supplementary file1 (DOCX 63 KB) [file 431_2023_5350_MOESM1_ESM.docx]

**Tracking of apolipoprotein B levels measured in childhood and adolescence: Systematic review and meta-analysis**

**European Journal of Pediatrics**

Oliver Stanesby, Zhen Zhou, Ricardo Fonseca, Tetsuhiro Kidokoro, Petr Otahal, Brooklyn J. Fraser, Feitong Wu , Markus Juonala, Jorma S.A. Viikari, Olli T. Raitakari, Grant R. Tomkinson, Costan G. Magnussen

Correspondence: Costan G. Magnussen – Baker Heart and Diabetes Institute; PO Box 6492, Melbourne Victoria 3004, Australia; +61 3 8532 1111; Costan.Magnussen@baker.edu.au

**Supplementary Table 1a. PRISMA 2020 for abstracts checklist.**

| **Section and Topic** | **Item** | **Checklist item** | **Reported, Yes/No** |
| --- | --- | --- | --- |
| **Title** |  |  |  |
| Title | 1 | Identify the report as a systematic review. | Yes |
| **Background** |  |  |  |
| Objectives | 2 | Provide an explicit statement of the main objective(s) or question(s) the review addresses. | Yes |
| **Methods** |  |  |  |
| Eligibility criteria | 3 | Specify the inclusion and exclusion criteria for the review. | Yes |
| Information sources | 4 | Specify the information sources (e.g. databases, registers) used to identify studies and the date when each was last searched. | Yes |
| Risk of bias | 5 | Specify the methods used to assess risk of bias in the included studies. | Yes |
| Synthesis of results | 6 | Specify the methods used to present and synthesise results. | Yes |
| **Results** |  |  |  |
| Included studies | 7 | Give the total number of included studies and participants and summarise relevant characteristics of studies. | Yes |
| Synthesis of results | 8 | Present results for main outcomes, preferably indicating the number of included studies and participants for each. If meta-analysis was done, report the summary estimate and confidence/credible interval. If comparing groups, indicate the direction of the effect (i.e. which group is favoured). | Yes |
| **Discussion** |  |  |  |
| Limitations of evidence | 9 | Provide a brief summary of the limitations of the evidence included in the review (e.g. study risk of bias, inconsistency and imprecision). | Yes |
| Interpretation | 10 | Provide a general interpretation of the results and important implications. | Yes |
| **Other** |  |  |  |
| Funding | 11 | Specify the primary source of funding for the review. | Yes (in article) |
| Registration | 12 | Provide the register name and registration number. | Yes |

Adapted from: Page MJ, McKenzie JE, Bossuyt PM, Boutron I, Hoffmann TC, Mulrow CD, et al. The PRISMA 2020 statement: an updated guideline for reporting systematic reviews. BMJ 2021;372:71. doi: 10.1136/bmj.n71

**Supplementary Table 1b. PRISMA 2020 checklist.**

| **Section and Topic** | **Item** | **Checklist item** | **Location where item is reported** |
| --- | --- | --- | --- |
| **Title** |  |  |  |
| Title | 1 | Identify the report as a systematic review. | Title |
| **Abstract** |  |  |  |
| Abstract | 2 | See the PRISMA 2020 for Abstracts checklist. | Appendix 1 |
| **Introduction** |  |  |  |
| Rationale | 3 | Describe the rationale for the review in the context of existing knowledge. | Paragraphs 1-2 |
| Objectives | 4 | Provide an explicit statement of the objective(s) or question(s) the review addresses. | Paragraph 2 |
| **Methods** |  |  |  |
| Eligibility criteria | 5 | Specify the inclusion and exclusion criteria for the review and how studies were grouped for the syntheses. | Eligibility criteria section, Table 1 |
| Information sources | 6 | Specify all databases, registers, websites, organisations, reference lists and other sources searched or consulted to identify studies. Specify the date when each source was last searched or consulted. | Information sources section |
| Search strategy | 7 | Present the full search strategies for all databases, registers and websites, including any filters and limits used. | Search strategy section, Appendix 2 |
| Selection process | 8 | Specify the methods used to decide whether a study met the inclusion criteria of the review, including how many reviewers screened each record and each report retrieved, whether they worked independently, and if applicable, details of automation tools used in the process. | Study selection section |
| Data collection process | 9 | Specify the methods used to collect data from reports, including how many reviewers collected data from each report, whether they worked independently, any processes for obtaining or confirming data from study investigators, and if applicable, details of automation tools used in the process. | Data collection section |
| Data items | 10a | List and define all outcomes for which data were sought. Specify whether all results that were compatible with each outcome domain in each study were sought (e.g. for all measures, time points, analyses), and if not, the methods used to decide which results to collect. | Data items and effect measures section, Table 1 |
|  | 10b | List and define all other variables for which data were sought (e.g. participant and intervention characteristics, funding sources). Describe any assumptions made about any missing or unclear information. | Data items and effect measures section |
| Study risk of bias assessment | 11 | Specify the methods used to assess risk of bias in the included studies, including details of the tool(s) used, how many reviewers assessed each study and whether they worked independently, and if applicable, details of automation tools used in the process. | Risk of bias assessment section |
| Effect measures | 12 | Specify for each outcome the effect measure(s) (e.g. risk ratio, mean difference) used in the synthesis or presentation of results. | Data items and effect measures section, Synthesis methods section, Table 1, Table 2 |
| Synthesis methods | 13a | Describe the processes used to decide which studies were eligible for each synthesis (e.g. tabulating the study intervention characteristics and comparing against the planned groups for each synthesis (item #5)). | Synthesis methods section, Table 2 footnotes |
|  | 13b | Describe any methods required to prepare the data for presentation or synthesis, such as handling of missing summary statistics, or data conversions. | Synthesis methods section |
|  | 13c | Describe any methods used to tabulate or visually display results of individual studies and syntheses. | Synthesis methods section |
|  | 13d | Describe any methods used to synthesize results and provide a rationale for the choice(s). If meta-analysis was performed, describe the model(s), method(s) to identify the presence and extent of statistical heterogeneity, and software package(s) used. | Synthesis methods section |
|  | 13e | Describe any methods used to explore possible causes of heterogeneity among study results (e.g. subgroup analysis, meta-regression). | Synthesis methods section paragraph 2 |
|  | 13f | Describe any sensitivity analyses conducted to assess robustness of the synthesized results. | Study characteristics results section |
| Reporting bias assessment | 14 | Describe any methods used to assess risk of bias due to missing results in a synthesis (arising from reporting biases). | Reporting bias assessment, Figure 4 caption |
| Certainty assessment | 15 | Describe any methods used to assess certainty (or confidence) in the body of evidence for an outcome. | Certainty of evidence assessment section |
| **Results** |  |  |  |
| Study selection | 16a | Describe the results of the search and selection process, from the number of records identified in the search to the number of studies included in the review, ideally using a flow diagram. | Study selection section, Figure 1 |
|  | 16b | Cite studies that might appear to meet the inclusion criteria, but which were excluded, and explain why they were excluded. | Figure 1 |
| Study characteristics | 17 | Cite each included study and present its characteristics. | Study selection section, Study characteristics section, Table 2, Table 3 |
| Risk of bias in studies | 18 | Present assessments of risk of bias for each included study. | Risk of bias section, Table 3 by cohort (by study available on request) |
| Results of individual studies | 19 | For all outcomes, present, for each study: (a) summary statistics for each group (where appropriate) and (b) an effect estimate and its precision (e.g. confidence/credible interval), ideally using structured tables or plots. | Table 2 |
| Results of syntheses | 20a | For each synthesis, briefly summarise the characteristics and risk of bias among contributing studies. | Risk of bias section, Results of syntheses section, Table 3 |
|  | 20b | Present results of all statistical syntheses conducted. If meta-analysis was done, present for each the summary estimate and its precision (e.g. confidence/credible interval) and measures of statistical heterogeneity. If comparing groups, describe the direction of the effect. | Results of syntheses section, Figures 2-3 |
|  | 20c | Present results of all investigations of possible causes of heterogeneity among study results. | Results of syntheses section |
|  | 20d | Present results of all sensitivity analyses conducted to assess the robustness of the synthesized results. | Study characteristics results section |
| Reporting biases | 21 | Present assessments of risk of bias due to missing results (arising from reporting biases) for each synthesis assessed. | Reporting biases section, Figure 4 |
| Certainty of evidence | 22 | Present assessments of certainty (or confidence) in the body of evidence for each outcome assessed. | Discussion paragraph 1,  Risk of bias results section, Study characteristics results section, Results of syntheses section, Table 3 |
| **Discussion** |  |  |  |
| Discussion | 23a | Provide a general interpretation of the results in the context of other evidence. | Discussion |
|  | 23b | Discuss any limitations of the evidence included in the review. | Strengths and limitations section, throughout discussion |
|  | 23c | Discuss any limitations of the review processes used. | Strengths and limitations section |
|  | 23d | Discuss implications of the results for practice, policy, and future research. | Discussion, Conclusions section |
| **Other information** |  |  |  |
| Registration and protocol | 24a | Provide registration information for the review, including register name and registration number, or state that the review was not registered. | Methods paragraph 1 |
|  | 24b | Indicate where the review protocol can be accessed, or state that a protocol was not prepared. | Methods paragraph 1 |
|  | 24c | Describe and explain any amendments to information provided at registration or in the protocol. |  |
| Support | 25 | Describe sources of financial or non-financial support for the review, and the role of the funders or sponsors in the review. | Sources of Funding section |
| Competing interests | 26 | Declare any competing interests of review authors. | Conflict of interest statement |
| Availability of data, code and other materials | 27 | Report which of the following are publicly available and where they can be found: template data collection forms; data extracted from included studies; data used for all analyses; analytic code; any other materials used in the review. | Methods section, Appendix 3, Data availability statement |

Adapted from: Page MJ, McKenzie JE, Bossuyt PM, Boutron I, Hoffmann TC, Mulrow CD, et al. The PRISMA 2020 statement: an updated guideline for reporting systematic reviews. BMJ 2021;372:71. doi: 10.1136/bmj.n71

**Supplementary Table 2a. MEDLINE (via Ovid) search strategy terms.**

| **#** | **Query** | **Results from 16 Oct 2023** |
| --- | --- | --- |
| 1 | exp apolipoproteins/ or apolipoprotein*.tw. or apo?b*.tw. or lipoprotein??b*.tw. or LPB.tw. | 75,452 |
| 2 | exp adolescent/ or exp child/ or exp infant/ or child*.tw. or adolescen*.tw. or youth*.tw. or infant*.tw. or infancy*.tw. or newborn*.tw. or pediatric*.tw. or paediatric*.tw. or pubert*.tw. or pre?pubert*.tw. or boy*.tw. or girl*.tw. | 4,705,167 |
| 3 | exp "correlation of data"/ or track*.tw. or persist*.tw. or correlat*.tw. or stable*.tw. or stabil*.tw. | 4,249,691 |
| 4 | 1 and 2 and 3 | 1,357 |

Ovid MEDLINE(R) ALL <1946 to 13 October 2023>.

**Supplementary Table 2b. Embase (via Ovid) search strategy terms.**

| **#** | **Query** | **Results from 16 October 2023** |
| --- | --- | --- |
| 1 | exp apolipoproteins/ or apolipoprotein*.tw. or apo?b*.tw. or lipoprotein??b*.tw. or LPB.tw. | 126,866 |
| 2 | exp adolescent/ or exp child/ or exp infant/ or child*.tw. or adolescen*.tw. or youth*.tw. or infant*.tw. or infancy*.tw. or newborn*.tw. or pediatric*.tw. or paediatric*.tw. or pubert*.tw. or pre?pubert*.tw. or boy*.tw. or girl*.tw. | 4,860,331 |
| 3 | exp "correlation of data"/ or track*.tw. or persist*.tw. or correlat*.tw. or stable*.tw. or stabil*.tw. | 5,461,875 |
| 4 | 1 and 2 and 3 | 1,870 |

Embase <1974 to 13 October 2023>.

**Supplementary Table 2c. Web of Science Core Collection search strategy terms.**

| **#** | **Query** | **Results from 16 October 2023** |
| --- | --- | --- |
| 1 | TS=(apolipoprotein* or apo?b* or apob* or lipoprotein?b* or lipoproteinb* or LPB) | 87,639 |
| 2 | TS=(child* or adolescen* or youth* or infant* or infancy* or newborn* or pediatric* or paediatric* or pubert* or pre?pubert* or boy* or girl*) | 3,378,150 |
| 3 | TS=(track* or persist* or correlat* or stable* or stabil*) | 8,504,965 |
| 4 | #1 AND #2 AND #3 | 786 |

Web of Science Core Collection (Editions: All) <1945 to 16 October 2023>.

**Supplementary Table 2d. Google Scholar search strategy terms.**

| **#** | **Query** | **Results from 16 Oct 2023** |
| --- | --- | --- |
| 1 | (apolipoprotein* OR apo b* OR apob* OR lpb) AND (child* OR adolescen* OR youth* OR infant* OR infancy* OR newborn* OR pediatric* or paediatric* or pubert* or boy* or girl*) AND (track* OR persist* OR correlat* OR stable* OR stabil*) | First 200 of 1270 sorted by relevance on 14 January 2022 + new records published since 2022 in first 200 of 1500 sorted by relevance on 16 October 2023 |

Google Scholar <no lower limit to 16 October 2023>.

**Supplementary Table 3. Summary of extracted data**

| **Cohort** | **Study (publication)** | **Lipid** | **Effect size, r** | **Sample size, n** | **Summary measure** | **Sex** | **Age at baseline, y** | **Length of follow-up, y** |
| --- | --- | --- | --- | --- | --- | --- | --- | --- |
| Bogalusa Heart Study | Bao W, Srinivasan S, Berenson G. Tracking of serum apolipoproteins A-I and B in children and young adults: The Bogalusa Heart Study. J Clin Epidemiol. 1993;46(7):609-16. | apoB | 0.61 | 241 | correlation (partial Spearman) | males | 5.5 | 3 |
|  |  | apoB | 0.65 | 280 | correlation (partial Spearman) | females | 5.5 | 3 |
|  |  | apoB | 0.57 | 135 | correlation (partial Spearman) | males | 5.5 | 3 |
|  |  | apoB | 0.59 | 158 | correlation (partial Spearman) | females | 5.5 | 3 |
|  |  | apoB | 0.64 | 233 | correlation (partial Spearman) | males | 11 | 3 |
|  |  | apoB | 0.53 | 237 | correlation (partial Spearman) | females | 11 | 3 |
|  |  | apoB | 0.6 | 144 | correlation (partial Spearman) | males | 11 | 3 |
|  |  | apoB | 0.62 | 133 | correlation (partial Spearman) | females | 11 | 3 |
|  |  | apoB | 0.2 | 36 | correlation (partial Spearman) | males | 16 | 4 |
|  |  | apoB | 0.44 | 52 | correlation (partial Spearman) | females | 16 | 4 |
|  |  | apoB | 0.53 | 29 | correlation (partial Spearman) | males | 16 | 4 |
|  |  | apoB | 0.59 | 50 | correlation (partial Spearman) | females | 16 | 4 |
|  |  | LDL-C ^b^ | 0.67 | 510 | correlation (partial Spearman) | males | 10.5 | 4 |
|  |  | LDL-C ^b^ | 0.67 | 308 | correlation (partial Spearman) | males | 10.5 | 4 |
|  |  | LDL-C ^b^ | 0.64 | 569 | correlation (partial Spearman) | females | 10.5 | 4 |
|  |  | LDL-C ^b^ | 0.65 | 341 | correlation (partial Spearman) | females | 10.5 | 4 |
|  |  | apoB ^a,b^ | 0.59 | 510 | correlation (partial Spearman) | males | 10.5 | 4 |
|  |  | apoB ^a,b^ | 0.58 | 308 | correlation (partial Spearman) | males | 10.5 | 4 |
|  |  | apoB ^a,b^ | 0.58 | 569 | correlation (partial Spearman) | females | 10.5 | 4 |
|  |  | apoB ^a,b^ | 0.59 | 341 | correlation (partial Spearman) | females | 10.5 | 4 |
|  | Srinivasan S, Sharma C, Foster T, Berenson G. Longitudinal changes of serum lipid and apoB levels in a newborn-infant cohort. Metabolism. 1982;31(2):167-71. | apoB | 0.53 | 30 | correlation (simple correlation, type unspecified) | both | 0 | 1 |
|  |  | apoB | 0.48 | 30 | correlation (simple correlation, type unspecified) | both | 0 | 2 |
|  |  | apoB | 0.4 | 30 | correlation (simple correlation, type unspecified) | both | 0 | 3 |
|  |  | apoB | 0.55 | 30 | correlation (simple correlation, type unspecified) | both | 0.5 | 1.5 |
|  |  | apoB | 0.7 | 30 | correlation (simple correlation, type unspecified) | both | 0.5 | 2.5 |
|  |  | apoB | 0.74 | 30 | correlation (simple correlation, type unspecified) | both | 1 | 1 |
|  |  | apoB | 0.71 | 30 | correlation (simple correlation, type unspecified) | both | 1 | 2 |
|  |  | apoB | 0.66 | 30 | correlation (simple correlation, type unspecified) | both | 2 | 1 |
| Cardiovascular Risk in Young Finns Study | Porkka K, Viikari J, Taimela S, Dahl M, Åkerblom H. Tracking and predictiveness of serum lipid and lipoprotein measurements in childhood: A 12-year follow-up. The Cardiovascular Risk in Young Finns Study. Am J Epidemiol. 1994;140(12):1096-110. | apoB ^a^ | 0.72 | 177 ^b^ | correlation (Spearman) | females | 15.9 | 3 |
|  |  | apoB ^a^ | 0.71 | 156 ^b^ | correlation (Spearman) | males | 15.4 | 3 |
| Four Provinces Study | del Villar-Rubín S, Escorihuela R, Ortega-Senovilla H, de Oya M, Garcés C. High tracking of apolipoprotein B levels from the prepubertal age to adolescence in Spanish children. Acta Paediatr. 2013;102(8):e374-e7. | apoB ^a,b^ | 0.607 | 179 | correlation (paired-sample) | males | 7.2 | 7.4 |
|  |  | apoB ^a,b^ | 0.466 | 206 | correlation (paired-sample) | females | 7.2 | 7.4 |
|  |  | LDL-C ^b^ | 0.472 | 179 | correlation (paired-sample) | males | 7.2 | 7.4 |
|  |  | LDL-C ^b^ | 0.339 | 206 | correlation (paired-sample) | females | 7.2 | 7.4 |
| LIFE Child Study | Maidowski L, Kiess W, Baber R, Dathan-Stumpf A, Ceglarek U, Vogel M. Tracking of serum lipids in healthy children on a year-to-year basis. BMC Cardiovasc Disord. 2023;23(1):386. | apoB ^a,c^ | 0.64 | 49 | Correlation (Pearson, between standard deviation score values) | both | 1 | 1 |
|  |  | apoB ^a,c^ | 0.78 | 43 | Correlation (Pearson, between standard deviation score values) | both | 2 | 1 |
|  |  | apoB ^a,c^ | 0.74 | 38 | Correlation (Pearson, between standard deviation score values) | both | 3 | 1 |
|  |  | apoB ^a,c^ | 0.81 | 44 | Correlation (Pearson, between standard deviation score values) | both | 4 | 1 |
|  |  | apoB ^a,c^ | 0.73 | 57 | Correlation (Pearson, between standard deviation score values) | both | 5 | 1 |
|  |  | apoB ^a,c^ | 0.80 | 65 | Correlation (Pearson, between standard deviation score values) | both | 6 | 1 |
|  |  | apoB ^a,c^ | 0.81 | 95 | Correlation (Pearson, between standard deviation score values) | both | 7 | 1 |
|  |  | apoB ^a,c^ | 0.78 | 110 | Correlation (Pearson, between standard deviation score values) | both | 8 | 1 |
|  |  | apoB ^a,c^ | 0.80 | 150 | Correlation (Pearson, between standard deviation score values) | both | 9 | 1 |
|  |  | apoB ^a,c^ | 0.83 | 156 | Correlation (Pearson, between standard deviation score values) | both | 10 | 1 |
|  |  | apoB ^a,c^ | 0.80 | 167 | Correlation (Pearson, between standard deviation score values) | both | 11 | 1 |
|  |  | apoB ^a,c^ | 0.79 | 185 | Correlation (Pearson, between standard deviation score values) | both | 12 | 1 |
|  |  | apoB ^a,c^ | 0.79 | 221 | Correlation (Pearson, between standard deviation score values) | both | 13 | 1 |
|  |  | apoB ^a,c^ | 0.81 | 151 | Correlation (Pearson, between standard deviation score values) | both | 14 | 1 |
|  |  | apoB ^a,c^ | 0.82 | 103 | Correlation (Pearson, between standard deviation score values) | both | 15 | 1 |
|  |  | apoB ^a,c^ | 0.77 | 85 | Correlation (Pearson, between standard deviation score values) | both | 16 | 1 |
|  |  | apoB ^a,c^ | 0.78 | 30 | Correlation (Pearson, between standard deviation score values) | both | 17 | 1 |
| Madrid Study (no official name) | Sánchez-Bayle M, Gonzalez-Requejo A, Ruiz-Jarabo C, Asensio J, Baeza J, Vila S, et al. Serum lipids and apolipoproteins in Spanish children and adolescents: A 5 year follow-up. Acta Paediatr. 1996;85(3):292-4. | apoB | 0.66 | 84 | correlation (type not specified) | both | 6 | 5 |
|  |  | apoB | 0.64 | 89 | correlation (type not specified) | both | 10 | 5 |
|  |  | apoB | 0.69 | 64 | correlation (type not specified) | both | 14 | 5 |
|  |  | apoB ^a,b^ | 0.66 | 237 | correlation (type not specified) | both | 10 | 5 |
|  |  | apoB | 0.68 | 119 | correlation (type not specified) | males | 10 | 5 |
|  |  | apoB | 0.66 | 118 | correlation (type not specified) | females | 10 | 5 |
|  |  | LDL-C | 0.62 | 84 | correlation (type not specified) | both | 6 | 5 |
|  |  | LDL-C | 0.61 | 89 | correlation (type not specified) | both | 10 | 5 |
|  |  | LDL-C | 0.73 | 64 | correlation (type not specified) | both | 14 | 5 |
|  |  | LDL-C ^b^ | 0.65 | 237 | correlation (type not specified) | both | 10 | 5 |
|  |  | LDL-C | 0.66 | 119 | correlation (type not specified) | males | 10 | 5 |
|  |  | LDL-C | 0.68 | 118 | correlation (type not specified) | females | 10 | 5 |
| Rivas-Vaciamadrid Study | Peláez Gómez de Salazar M, Sánchez Bayle M, González-Requejo A, Ruiz-Jarabo Quemada C, Asensio Antón J, Otero de Becerrea J. Perfil lipídico en niños de Rivas-Vaciamadrid. Seguimiento de 2 años. An Esp Pediatr. 2000;52(5):443-6. | apoB ^a,b^ | 0.63 | 493 | correlation (Pearson) | both | 6 | 2 |
|  |  | LDL-C ^b^ | 0.69 | 493 | correlation (Pearson) | both | 6 | 2 |
|  | Sánchez-Bayle M, Sánchez Bernardo A, Asensio Antón J, Ruiz-Jarabo Quemada C, Baeza Mínguez J, Morales San José M. Seguimiento de 5 años del perfil lipídico en los niños. Estudio Rivas-Vaciamadrid. An Esp Pediatr. 2005;63(1):34-8. | apoB ^a,b^ | 0.588 | 281 | correlation (Pearson) | both | 6 | 5 |
|  |  | apoB | 0.453 | 149 | correlation (Pearson) | males | 6 | 5 |
|  |  | apoB | 0.685 | 132 | correlation (Pearson) | females | 6 | 5 |
|  |  | LDL-C ^b^ | 0.649 | 281 | correlation (Pearson) | both | 6 | 5 |
|  |  | LDL-C | 0.556 | 149 | correlation (Pearson) | males | 6 | 5 |
|  |  | LDL-C | 0.711 | 132 | correlation (Pearson) | females | 6 | 5 |
| Tallinn Young Family Study | Kurvinen E, Aasvee K, Zordania R, Jauhiainen M, Sundvall J. Serum lipid and apolipoprotein profiles in newborns and six‐year‐old children: The Tallinn Young Family Study. Scand J Clin Lab Invest. 2005;65(7):541-50. | apoB ^a^ | 0.43 | 40 | correlation (Pearson) | both | 0 | 6 |
| Umeå Study (no official name) | Öhlund I, Hernell O, Hörnell A, Lind T. Serum lipid and apolipoprotein levels in 4-year-old children are associated with parental levels and track over time. Eur J Clin Nutr. 2011;65(4):463-9. | apoB ^a^ | 0.35 | 58 | correlation (Pearson) | males | 0.5 | 1 |
|  |  | apoB ^a,b^ | 0.55 | 60 | correlation (Pearson) | females | 0.5 | 1 |
|  |  | apoB ^a^ | 0.33 | 47 | correlation (Pearson) | males | 0.5 | 3.5 |
|  |  | apoB ^a,b^ | 0.58 | 57 | correlation (Pearson) | females | 0.5 | 3.5 |
|  |  | apoB ^a,b^ | 0.51 | 47 | correlation (Pearson) | males | 1 | 3 |
|  |  | apoB ^a,b^ | 0.68 | 57 | correlation (Pearson) | females | 1 | 3 |
|  |  | apoB ^a,b^ | 0.56 | 47 | correlation (Pearson) | males | 1.5 | 2.5 |
|  |  | apoB ^a,b^ | 0.65 | 57 | correlation (Pearson) | females | 1.5 | 2.5 |
|  |  | LDL-C |  | 58 | correlation (Pearson) | males | 0.5 | 1 |
|  |  | LDL-C ^b^ | 0.36 | 60 | correlation (Pearson) | females | 0.5 | 1 |
|  |  | LDL-C |  | 47 | correlation (Pearson) | males | 0.5 | 3.5 |
|  |  | LDL-C ^b^ | 0.39 | 57 | correlation (Pearson) | females | 0.5 | 3.5 |
|  |  | LDL-C ^b^ | 0.3 | 47 | correlation (Pearson) | males | 1 | 3 |
|  |  | LDL-C ^b^ | 0.57 | 57 | correlation (Pearson) | females | 1 | 3 |
|  |  | LDL-C ^b^ | 0.48 | 47 | correlation (Pearson) | males | 1.5 | 2.5 |
|  |  | LDL-C ^b^ | 0.51 | 57 | correlation (Pearson) | females | 1.5 | 2.5 |

apoB: apolipoprotein B; LDL-C: low-density lipoprotein cholesterol; ^a^ Used to derive summary estimate of study for main meta-analysis (Figure 2); ^b^ Used to derive summary estimate of study for meta-analysis of tracking of apoB vs. LDL-C (Figure 2); ^c^ Calculated by multiplying the proportion of females or males in the sample by the size of the sample in the apoB tracking analysis.

| Study (article abbreviation): |  |
| --- | --- |
| Assessor: |  |
| Date: |  |

**Risk of Bias Assessment**

**Selection bias**

**1) Representativeness of the baseline^†^ sample**

★ the individuals selected to participate in the baseline study were likely to be representative of the population that was targeted by the sampling frame

the individuals selected to participate in the baseline study were not likely to be representative of the population that was targeted by the sampling frame

no or insufficient description of the selection of the baseline sample to determine representativeness

**Attrition bias**

**2) Adequacy of follow-up of the baseline cohort(s)**

Select all that apply (note: maximum 1 point awarded for each question with at least one ★ category selected)

★ ≤ 20% lost to follow-up ^§^

★ > 20% lost to follow-up AND those lost to follow-up were comparable to those retained or used a

statistical approach that attempted to account for possible differential loss to follow-up

> 20% lost to follow-up AND those lost to follow-up were not comparable to those retained AND no statistical attempt to account for possible differential loss to follow-up

> 20% lost to follow-up AND no description of those lost to follow-up

no or insufficient detail to determine adequacy of follow-up

**Measurement bias**

**3) Protocols for measurement of apoB/LDL-C levels at baseline and follow-up(s)**

★ measured using protocols that met relevant quality assurance standards/certification

(e.g., laboratory(ies) met or participate in an established external quality assurance program or followed

standardised procedures)

did not measure using protocols that met relevant quality assurance standards/certification

no or insufficient description to determine measurement protocols

**4) Multiple measurements used to define baseline and follow-up apoB/LDL-C levels**

★ baseline AND follow-up apoB/LDL-C levels each determined by more than one measurement

baseline or follow-up apoB/LDL-C levels determined by only one measurement

no or insufficient detail to determine the number of measurements used to define apoB/LDL-C levels

**5) Fasting status of participants at baseline and follow-up serum sampling**

★ participants were fasted or instructed to be fasted for serum sampling at baseline and follow-up(s)

participants were not fasted or instructed not to be fasted for serum sampling at baseline or follow-up(s)

no or insufficient description to determine fasting status of participants at baseline and follow-up serum

sampling

**Confounding bias**

**6) Potential for confounding thought to impact apoB tracking estimates (length of follow-up)**

★ length of follow-up varied by ≤ 20% within the sample used for each tracking estimate

★ length of follow-up varied by > 20% within the sample used for a tracking estimate AND used a statistical approach that accounted for differences in length of follow-up

length of follow-up varied by > 20% within the sample used for a tracking estimate AND no statistical attempt to account for differences in length of follow-up

no or insufficient detail to determine variation in length of follow-up and whether study accounted for differences in length of follow-up

**7) Potential for confounding thought to impact apoB tracking estimates (age at baseline, sex)**

★ the sample was all-male or all-female OR used a statistical approach that accounted for sex AND age at

baseline varied by ≤ 20% of the maximum age at baseline OR used a statistical approach that accounted for

age at baseline

the sample comprised both males and females AND no statistical attempt to account for sex

OR age at baseline varied by > 20% of the maximum age at baseline AND no statistical attempt to account for age at baseline

no or insufficient detail to determine sex composition of sample, variation in age at baseline and whether

study accounted for differences in sex and age at baseline

**Conflict of interest bias**

**8) Risk of bias due to conflicts of interest**

★ declarations of conflict of interest or identification of funding sources and their role in the study and

article AND professional judgment and actions regarding the study and article were unlikely to be influenced by a secondary interest

no declarations of conflict of interest or identification of funding sources and their role in the study and

article

professional judgment or actions regarding the study or article were likely to be influenced by a secondary

interest

**Comments**

|  |
| --- |

**References**

Kristman V, Manno M, Côté P. Loss to follow-up in cohort studies: how much is too much? Eur J Epidemiol. 2004;19(8):751-60. doi: 10.1023/b:ejep.0000036568.02655.f8. PMID: 15469032.

**Risk of bias assessment – Instructions**

- Complete the tool separately for each study
- Use information from the article and supplement/appendices only, not external sources.
- Aspects relating to confounding, multiple measurements and 'estimates' apply to the estimates we examine in our meta-analyses: tracking of continuous variables of apoB (and LDL-C, if also measured/analysed) – defined in eligibility criteria.
- Please use the comments box to document justifications for selection.
- Scoring: A maximum of one point is awarded for each question with one or more ★ categories selected)
- Scoring for item 5: One point is awarded if LDL-C tracking was not measured regardless of whether the sample was fasted. No points are awarded if LDL-C tracking was measured but the sample was not fasted.
